# Characterization of Pharmaceutical Transformation Products by High-Field Asymmetric Waveform Ion Mobility and Infrared Ion Spectroscopy Coupled to Mass Spectrometry

**DOI:** 10.1021/jasms.5c00039

**Published:** 2025-05-12

**Authors:** César A. G. Dantas, Pedro H. M. Garcia, Thiago C. Correra

**Affiliations:** Department of Fundamental Chemistry, Institute of Chemistry, 153989 University of São Paulo, Av. Prof. Lineu Prestes, 748, Cidade Universitária, São Paulo, São Paulo 05508-000, Brazil

**Keywords:** forced degradation, transformation products, mass spectrometry, ion mobility, infrared ion spectroscopy

## Abstract

The identification of drug degradation products is crucial for pharmaceutical development and quality control, as drug transformation products can significantly affect therapeutic efficacy and patient safety. Traditional analytical methods, such as high-performance liquid chromatography (HPLC) and *tandem* mass spectrometry (MS/MS), often require reference standards for accurate identification and may be unsuitable for resolving isomeric and isobaric degradation products. This study explores the use of high-field asymmetric waveform ion mobility spectrometry (FAIMS) and infrared multiple photon dissociation (IRMPD) spectroscopy coupled with mass spectrometry (MS) as an effective alternative for identifying drug degradation products without the need for previous chromatographic stages or the use of reference standards. Cyclophosphamide, a widely used DNA-alkylating agent in cancer and autoimmune therapies, is employed as a model system for this study. FAIMS enabled the separation of species based on their differential mobility, while IRMPD provided distinctive spectral data, allowing precise reference-standard-free structural elucidation. This integrated approach offers a robust solution for the identification of complex degradation products, advancing stability studies, formulation development, and quality control in pharmaceutical analysis.

## Graphical abstract

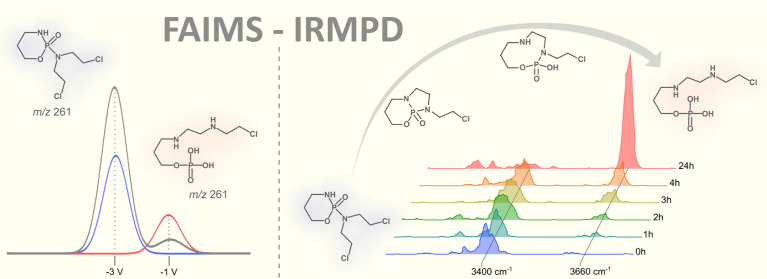

## Introduction

Pharmaceutical studies of forced chemical degradation represent an essential stage in the development of more stable drug formulations and ideal storage conditions for both raw materials and final formulations.^1^ These studies also allow for the evaluation of potential toxic agents and consequent risks for patient health ensuring the safety and therapeutic efficacy of these chemical species.^2^ The evaluation of degradation products of pharmaceutical ingredients must be evaluated under the influence of several factors, including humidity, pH, and exposure to temperature, light, and oxidizing agents, following the standards and guidelines established by regulatory agencies.^3–5^

The analytical methods employed for the identification of degradation products depend on the chemical nature of the species involved and the specific objectives of the analysis. Different spectroscopic and chromatographic techniques are often used for qualitative and quantitative analysis of transformation products generated in drug stability studies.^6,7^ Most studies indicate liquid chromatography coupled with UV–vis detectors (LC-UV) or *tandem* mass spectrometry (LC-MS/MS) as the techniques of choice for the analysis of complex matrices.^2,8,9^ However, these methodologies are limited when isomers or isobars, often generated in these forced degradations studies,^10–14^ need to be identified, as they may show similar chromatographic behavior and fragmentation patterns that hinder or prevent their differentiation.^15^

Cyclophosphamide (CP; Figure 1), a DNA alkylating agent used for the treatment of various types of neoplasms and autoimmune disorders, is a relevant example and model in this context.^16,17^ This nitrogen mustard-type prodrug is activated by the hepatic microsomal system, cytochrome P-450,^18,19^ and produces two main bioactive metabolites: (i) phosphoramide mustard, which has cytotoxic activity against cancer cells, and (ii) acrolein, a secondary metabolite involved in the main adverse events of cyclophosphamide^20–22^ (Figure 1a).

**Figure 1:**
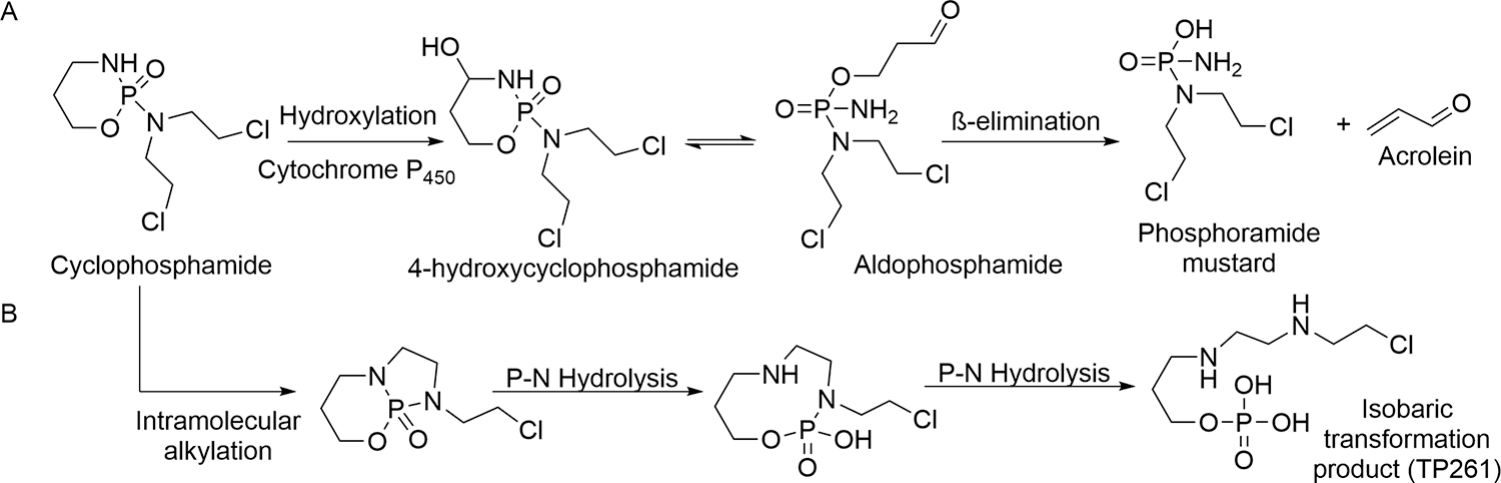
Cyclophosphamide structure and reactivity involved in its (A) activation route and in the (B) formation of isobaric transformation product TP261.

Although playing a relevant role in the activation of the prodrug, the reactivity of CP makes it necessary that special care should be taken during the handling and storage stages of CP formulations, since susceptible hydrolysis reactions and other processes can significantly affect the integrity of the cyclophosphamide molecule.^23,24^

These processes result in a decrease or loss of its pharmacological efficacy and possible undesirable side effects for patients, such as a decrease in the interaction between vitamin B12 and its target protein.^25,26^ For these reasons, many forced chemical degradation studies^2,27–29^ were dedicated to verify the conditions needed to guarantee the quality of CP during its production, transportation, and storage.^30–32^

Investigations into the possible mechanisms of chemical hydrolysis of cyclophosphamide have shown that decreasing the pH of the medium significantly increases the decomposition of the molecule and consequently the loss of pharmacological activity. This process was also shown to be accelerated by increasing the medium temperature.^26,33^ It has also been reported that CP can generate an isobaric transformation product that was previously identified by Gilard and co-workers^26,34^ as 3-({2-[(2-chloroethyl)­amino]­ethyl}­amino)­propyl dihydrogen phosphate (Figure 1b, TP261), a secondary degradation product generated after the cyclophosphamide molecule undergoes intramolecular alkylation, followed by subsequent hydrolysis of the P–N bonds.

Therefore, cyclophosphamide is an interesting model for evaluating forced chemical degradation approaches and related methods for the identification of isomeric and isobaric degradation products.

Ion mobility spectrometry (IMS) techniques coupled with mass spectrometry have stood out as a powerful methodology for separating and analyzing chemical species that share the same *m*/*z* in a complex matrix, a fact that is reflected in the growth in the number of studies presenting new applications of this technique in different areas.^15,35,36^

Despite the many flavors of ion mobility available, this technique can be described as a method to differentiate ions in the gas phase according to their geometries and shapes, as they modulate the ion interactions with a buffer gas.

More specifically, high-field asymmetric waveform ion mobility spectrometry (FAIMS) has emerged as an alternative to separate and analyze ion populations under atmospheric pressure conditions. This advantage allows FAIMS to be easily integrated with commercial mass spectrometers by setting up the FAIMS cell before the spectrometer inlet without extensive modifications.^37,38^ By allowing the differentiation of isobars and isomers, this approach could be easily employed as a more direct and powerful alternative for the qualitative analysis of the forced chemical degradation products.

One disadvantage of this approach would be that the identification of isomers by FAIMS usually depends on the analysis of reference standards, as the nature of species being evaluated by this technique cannot be determined *a priori* as their separation depends on a complex equilibrium between collisions and clustering and declustering of the ions with the buffer gas.^39,40^

In this context, infrared spectral data obtained in the gas phase through infrared multiple photon dissociation (IRMPD) spectroscopy could be used as a tool for the structural determination of FAIMS separated species (Figure 2).^41–43^

**Figure 2:**
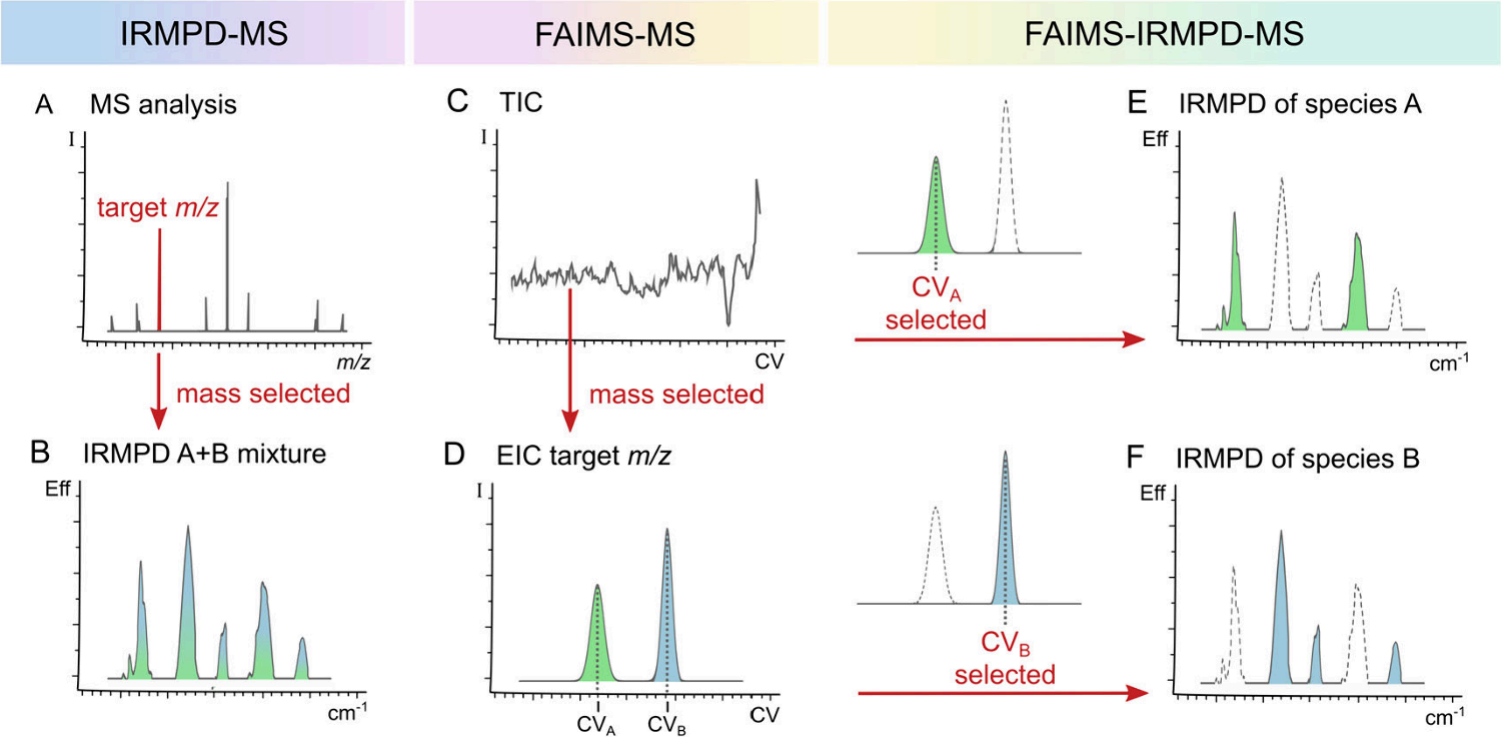
(A) Representation of a MS analysis of and subsequent analysis of a mixture of isobaric/isomeric ions A and B by (B) IRMPD spectrocopy. (D) FAIMS-MS can be achieved by mass selecting the ions reaching the detector as a function of CV as the total ion chromatogram (TIC) (C). Isomer specific FAIMS-IRMPD-MS for the isolated species A (E) and B (F).

This approach consists of promoting the photodissociation of a target ion in the gas phase by a tunable infrared radiation source and correlating the photofragmentation extension to the absorption bands of the desired ion. These absorptions can be directly compared to theoretical predictions of the IR spectra of probable species,^44^ allowing for their structural elucidation. Therefore, this would be a complementary technique to FAIMS, as it would allow the direct identification of the unknown species separated by the ion mobility stage.^37,45,46^

Therefore, we considered that, if both approaches were included in drug stability study protocols, a definitive reference standard free characterization of degradation products could be achieved providing valuable information and contributing to the development of more stable and effective pharmaceutical products,^47,48^ as well as to their quality control tests.^49–51^

In view of this information, the aim of this work was to analyze the isobaric transformation product of cyclophosphamide formed after forced chemical degradation by acid hydrolysis as a proof-of-concept system for evaluating the synergic use of FAIMS and IRMPD techniques coupled with mass spectrometry as a tool for isobaric and isomeric differentiation in drug degradation studies.

## Methodology

### Chemicals and Reagents

Cyclophosphamide monohydrate (CAS: 6055-19-2) was purchased from Sigma-Aldrich (San Luis, Mo., USA) and kept under refrigeration at 4 °C. The solutions subjected to the forced chemical degradation process were prepared using type I (ultrapure) water acidified with hydrochloric acid (0.1 N). The solutions analyzed by FAIMS and IRMPD were prepared using chromatographic grade methanol from BioScie (Anápolis, Brazil) and type I water.

### Degradation Studies

The degradation studies were conducted in accordance with the guidelines established by the International Council for Harmonization of Technical Requirements for Pharmaceuticals for Human Use (ICH) Q1A (R2).^52^ For the forced chemical degradation of cyclophosphamide by acid hydrolysis, 5 mg of the drug was solubilized in 5 mL of a 0.1 N HCl solution, providing a final solution of cyclophosphamide at a concentration of 3.10^–3^ M. This solution was then transferred to a reaction flask and heated under reflux at 70 °C for a period of 3 h. For control purposes, this procedure was repeated under the same hydrolysis conditions in a second forced chemical degradation test but with an extended heating period of 24 h. During this test, five aliquots were collected: one before heating (0 h), four at hourly intervals (1, 2, 3, and 4 h), and a final aliquot at 24 h. After the degradation tests, all samples were cooled and stored at 4 °C until analysis.

### Mass Spectrometry Analysis

For the MS and FAIMS/IRMPD analysis, aliquots from the degradation tests were diluted 100 and 10 times, respectively, in a MeOH:H_2_O (1:1 in volume) mixture and evaluated in positive ion mode by a modified AmaZon SL 3D ion trap mass spectrometer (Bruker Daltonics, Billerica, MA, USA) using a nanoESI (nESI) source.^53–55^ The nESI emitters (5–10 μm ID) were prepared in-house from borosilicate glass capillaries as described elsewhere.^37,53,56^ The voltage applied to the capillary was 3.0 kV, and the fragment ions observed in the MS^2^ spectra were generated by collision induced dissociation (CID) using Helium 5.0 (Air Products) as the collision gas. Distinct relative collision energies were applied based on each species’ dissociation threshold, and their values are reported alongside the corresponding mass spectra.

### FAIMS Analysis

FAIMS analyses were carried out by a commercial FAIMS apparatus (Heartland MS, KS, USA).^56,57^ The FAIMS cell was coupled to the standard MS inlet by a 3D-printed adaptor. The analysis were carried out with a curtain plate voltage of 1 kV, a nitrogen gas flow rate of 5 L/min, and a dispersion voltage of 4.5 kV. To avoid ion rejection at the grounded MS inlet during the scan, a 30 V bias was applied to the FAIMS electrodes. The compensation voltage (CV) was scanned from −20 to +20 V in 10 min (Figure S1), providing a scanning rate of 4 V/min. Lower scanning rates down to 1 V/min were tested but showed no resolution improvement over this higher rate. Despite the extended CV range explored, only the −5 to 0 V range is shown here, as no other populations other than cluster ions at CV −9.0 V were observed. These cluster ions had their nature confirmed by the protocol suggested by Glish and co-workers^58^ and by observing their CID pattern that revealed fragments common to both populations. The raw MS data (hollow points shown) were fitted with Gaussian functions and aligned to account for CV variations using Origin 2024.^59^

CID and IRMPD experiments were conducted during the FAIMS analysis by mass selecting and activating the target ions during the CV scan. Experiments for obtaining either the CID pattern or IRMPD spectra of specific populations were achieved by setting specific CV values, allowing a single ion population to reach the MS analyzer.

### Infrared Ion Spectroscopy

The AmaZon SL used in this work has modifications allowing a tunable infrared radiation beam in the 2800–3800 cm^–1^ range produced by a Nd:YAG pumped (10 Hz, Continuum Surelite II Milpitas, CA, USA) optical parametric oscillator/optical parametric amplifier, OPO/OPA (Laservision, USA), to reach the isolated target ions in the ion trap, promoting their photofragmentation.^53–55^ A total of 20 pulses (2 s of laser irradiation time) were used to allow for the acquisition of the IRMPD data reported. The IRMPD spectra were obtained by calculating the photofragmentation efficiency Eff from the photodissociation spectra as Eff = −ln­[*I*_P_/(*I*_P_ + Σ*I*_F_)], where *I*_P_ and Σ*I*_F_ are the intensity of the precursor ion and the sum of the intensities of the fragment ions, respectively. The raw photofragmentation data (data points on the IRMPD spectra) was smoothed using the Savitizky-Golay method with no boundary condition, polynomial order 2, and a 10 to 20 points window using Origin 2024.^59^

### Computational Methods

Simulated absorption spectra were calculated using the Gaussian 16 (Revision *C.01*)^60^ computational package employing the hybrid B3LYP functional^61^ for optimizations and frequency calculations at the 6-31++G­(d,p) basis set at 293.15 K as suggested in literature.^44^ The initial geometries and protonation sites used for the CP and TP261 calculations were based on chemical intuition and previous reports.^15^ Vibrational analysis showed the absence of imaginary frequencies in the species reported in this work and was compared to the experimental IRMPD spectra by using a scale factor of 0.956.^44^

## Results and Discussion

### Mass Spectrometry Results

The cyclophosphamide transformation products generated after the forced chemical degradation tests by acid hydrolysis were initially analyzed by MS and MS/MS in positive ion mode. Figure 3A,B shows the spectra obtained before and after the cyclophosphamide solution was exposed to heating for 3 h in the presence of 0.1 N HCl. As expected, when comparing the spectral data of these solutions, a decrease in the intensity of the protonated molecular ion with *m*/*z* 261 ([M + H]^+^) was observed due to the consumption of cyclophosphamide during the degradation test with the consequent formation of the primary and secondary transformation products. Based on the full scan analysis (Figure 3A,B), fragmentation patterns, and comparison of the data obtained with that found in scientific literature,^26,34,62^ 10 transformation products were identified. Their nature and putative composition of their fragments are presented in the Supporting Information (Table S1).

**Figure 3:**
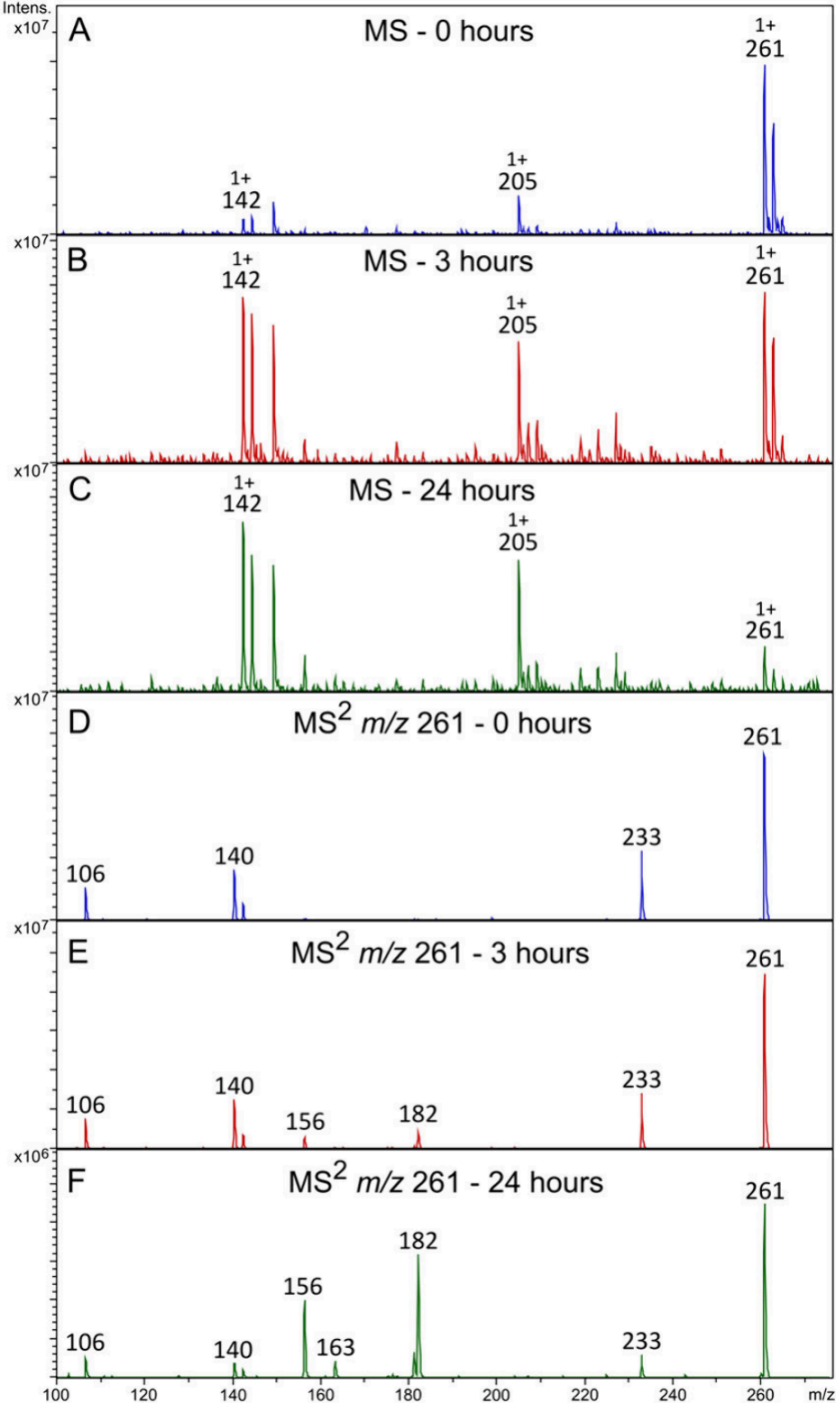
Full MS spectra for a CP solution (A) before (0 h) and after being submitted to forced chemical degradation by acid hydrolysis (0.1 N HCl) for (B) 3 h and (C) 24 h, and MS^2^ spectra (45% collision energy) of the *m*/*z* 261 ion (D) before (0 h) and after (E) 3 h and (F) 24 h of forced degradation.

To check for the possibility of isobaric transformation products, MS^2^ analyses of the ion with *m*/*z* 261 were carried out by CID. In the initial CP sample not exposed to heat, the formation of ions with *m*/*z* 233, *m*/*z* 142, *m*/*z* 140, and *m*/*z* 106 was observed (Figure 3D), consistent with previous results from the literature.^15^ However, for the cyclophosphamide solution heated for 3 h (Figure 3E), in addition to the fragmentation products described above, two other ions were observed, one with a higher intensity at *m*/*z* 182 and the other with a lower intensity at *m*/*z* 156. This evidence suggests that the degradation test promoted the formation of one or more isobaric cyclophosphamide chemical species.

### Ion Mobility of Degradation Products

To separate the possible populations of ions with *m*/*z* 261 present in the cyclophosphamide solution heated for 3 h, FAIMS-MS analyses were carried out. A range of compensation voltages (CV) from −20 to +20 V was scanned at a rate of 4 V/min, resulting in an extracted ion chromatogram (EIC) with two peaks referring to two populations of ions with *m*/*z* 261 (Figure 4B) in contrast to just one population observed for the initial CP solution (Figure 4A).

**Figure 4:**
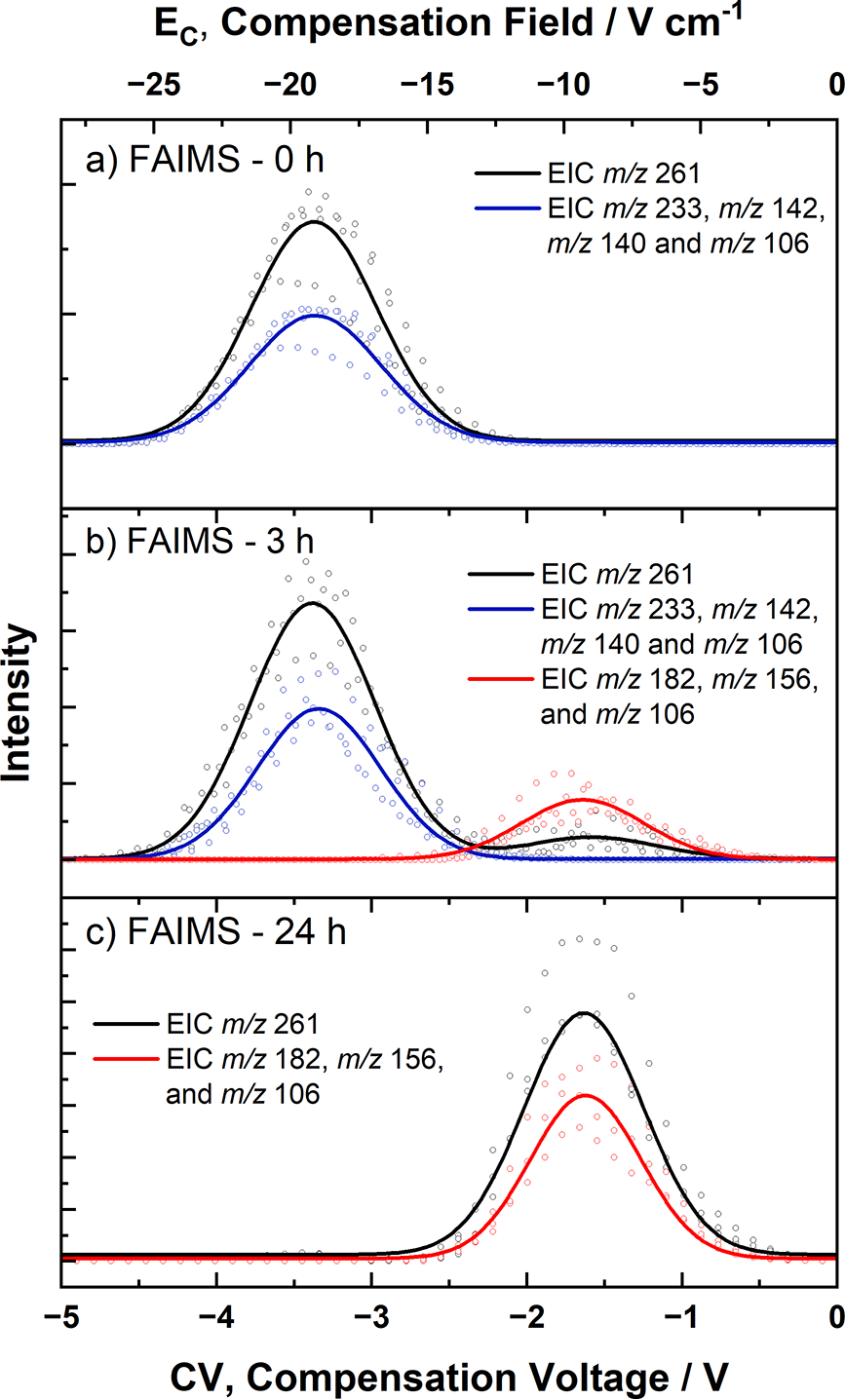
FAIMS spectrum for the CP solution submitted to forced chemical degradation at (A) 0, (B) 3, and (C) 24 h. Black traces indicate the extracted ion intensity for the ions with *m*/*z* 261, blue traces, the summed intensity of the ions with *m*/*z* 233, *m*/*z* 142, *m*/*z* 140, and *m*/*z* 106, and red traces, the summed intensity of the ions with *m*/*z* 182, *m*/*z* 156, and *m*/*z* 106 obtained for MS^2^ of the ion with *m*/*z* 261 employing 48% of collision energy in A and B and 21% in C.

The first population of ions presented significantly higher abundance and was detected in a CV range from −4 to −2 V, exhibiting maximum intensity at −3.4 V, while the second population of ions was observed in the CV range of −2.5 to −0.5 V, with maximum intensity at −1.5 V. Although there may be differences in the ionization efficiencies of distinct species,^63,64^ the relative peak areas can be considered approximate indications of their population distribution.^56^

New scans under the same parameters and CV range were carried out, while the fragmentation pattern was evaluated by MS^2^ to determine the fragmentation products of each of the ion populations detected. When one compared the MS^2^ spectra acquired from each one of the populations, different fragmentation profiles were revealed. Figure 4 shows that, while the first population promoted the formation of ions with *m*/*z* 233, *m*/*z* 142, *m*/*z* 140, and *m*/*z* 106 (Figure 4B – blue trace), the second population generated fragments with *m*/*z* 182, *m*/*z* 156, and *m*/*z* 106 (Figure 4B – red trace). These analyses allowed us to infer that the first population observed was the prodrug cyclophosphamide, due to the fragmentation pattern shown being similar to the one previously observed for [CP + H]^+^ and in Figure 4A, while the second population was attributed to an isobaric transformation product produced after the forced chemical degradation test.^15,34^

### Ion Spectroscopy of Specific Populations

The IRMPD experiments were carried out for the solution of the cyclophosphamide not exposed to heating and acid treatment (0 h) and for the solution resulting from the 3 h forced chemical degradation test. In the IRMPD spectrum acquired for the initial CP sample, shown in Figure 5A, one major band was observed at 3400 cm^–1^ and a minor absorption, at 3650 cm^–1^, in accordance to B3LYP/6-31++G­(d,p) simulations and previous results that assign the 3400 cm^–1^ band to the endocyclic nitrogen N---H oscillator at 3440 cm^–1^ and the 3650 cm^–1^ absorption to the protonated phosphate group ^+^H---O=P stretch predicted at 3636 cm^–1^.^15^ It should be noted that this ^+^H---O=P band also presented a low fragmentation efficiency in our previous studies.^15^

**Figure 5:**
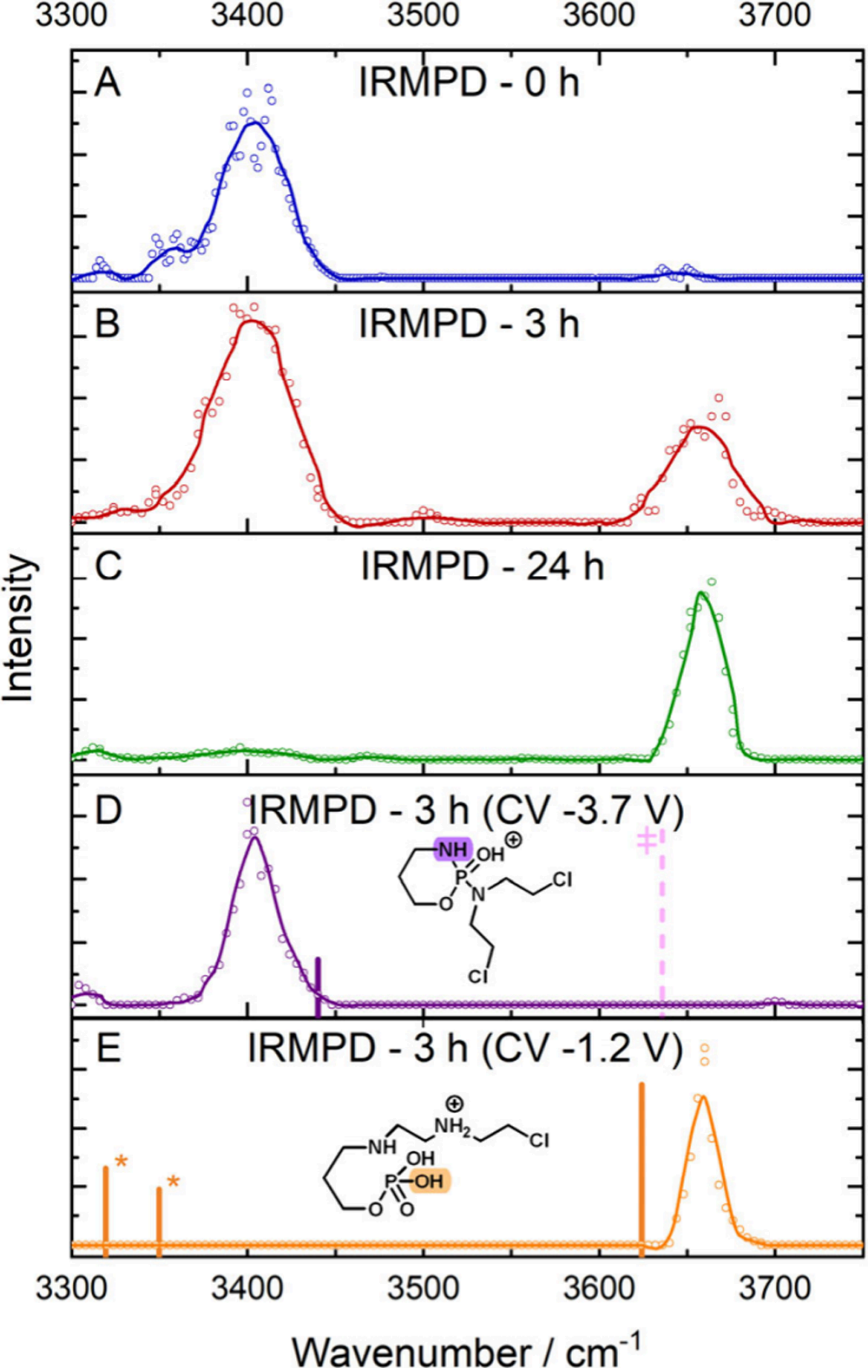
IRMPD spectrum for the CP solution submitted to forced chemical degradation at (A) 0 h, (B) 3 h, and (C) 24 h. (D, E) IRMPD spectra of the FAIMS selected populations at CV −3.7 and −1.2 V, in the CP solution submitted to forced chemical degradation at 3 h. Vertical lines indicate the predicted absorption bands at the B3LYP/6-31++G­(d,p) level of theory for the (D) protonated CP and (E) protonated TP261. ‡ indicates the low intensity OH band observed experimentally in previous studies (see ref 15 for details). * indicates bands with intensities lower than 50 km/mol that usually are not observed in the IRMPD spectra.^65^

For the solution that was subjected to hydrolysis for 3 h (Figure 5B), an absorption band at 3660 cm^–1^ was easily observed to be slightly blue-shifted from the one previously described for the protonated CP at 3650 cm^–1^ (Figure 5A).

Considering the previous studies from Gilard and co-workers^26,34^ that identified TP261 as an isobaric transformation product of CP using NMR, the vibrational spectra of the protonated TP261 was simulated at B3LYP/6-31++G­(d,p) level of theory. This simulation revealed that protonated TP261 shows one major absorption at 3625 cm^–1^ correlated to the neutral H---O–P stretch that could be assigned to the band observed experimentally at 3660 cm^–1^.

This simulation also shows N---H stretches at 3350 and 3319 cm^–1^ but with predicted intensities of 14 and 50 km/mol, respectively, that are usually below the intensity detection threshold of the IRMPD spectra.^65^ Therefore, the presence of a band in the N---H stretch in Figure 5B suggests the spectral features of CP can be overlapped with the absorption bands of the transformation product

To allow the acquisition of a clear spectrum without overlapping signals, the spectra of the two isolated ion populations with *m*/*z* 261 at distinct CV values were acquired by employing FAIMS-IRMPD-MS. Initially, the first population was selected by setting the CV to −3.7 V, so that only the first population of ions with *m*/*z* 261, referring to the prodrug cyclophosphamide, could reach the MS system and have its IRMPD spectrum acquired. The second population of ions with *m*/*z* 261, related to the isobaric transformation product, was selected by setting the CV to −1.2 V. These CV values were chosen to minimize the contribution of the other population, despite the good separation obtained.

When comparing the IRMPD spectra obtained for the two selected ion populations (Figure 5D,E), it is possible to note the presence of the band at 3400 cm^–1^ assigned to the CP endocyclic N---H stretch for the first population at CV −3.7 V (Figure 5D), which was not detected in the spectrum of the second population (Figure 5E). This allows us to confirm the absence of the endocyclic NH in the chemical structure of the isobaric transformation product produced and the lack of the low intensity NH bands predicted for TP261, suggesting this species is present as the isobaric transformation product present in the second FAIMS population.

### Photodepletion Experiments

To confirm that the different absorption bands observed for each population in the IRMPD spectra acquired in Figure 5 can be used to differentiate these species, the sample produced by forced degradation after 3 h was subjected to photodepletion experiments (Figure 6).

**Figure 6:**
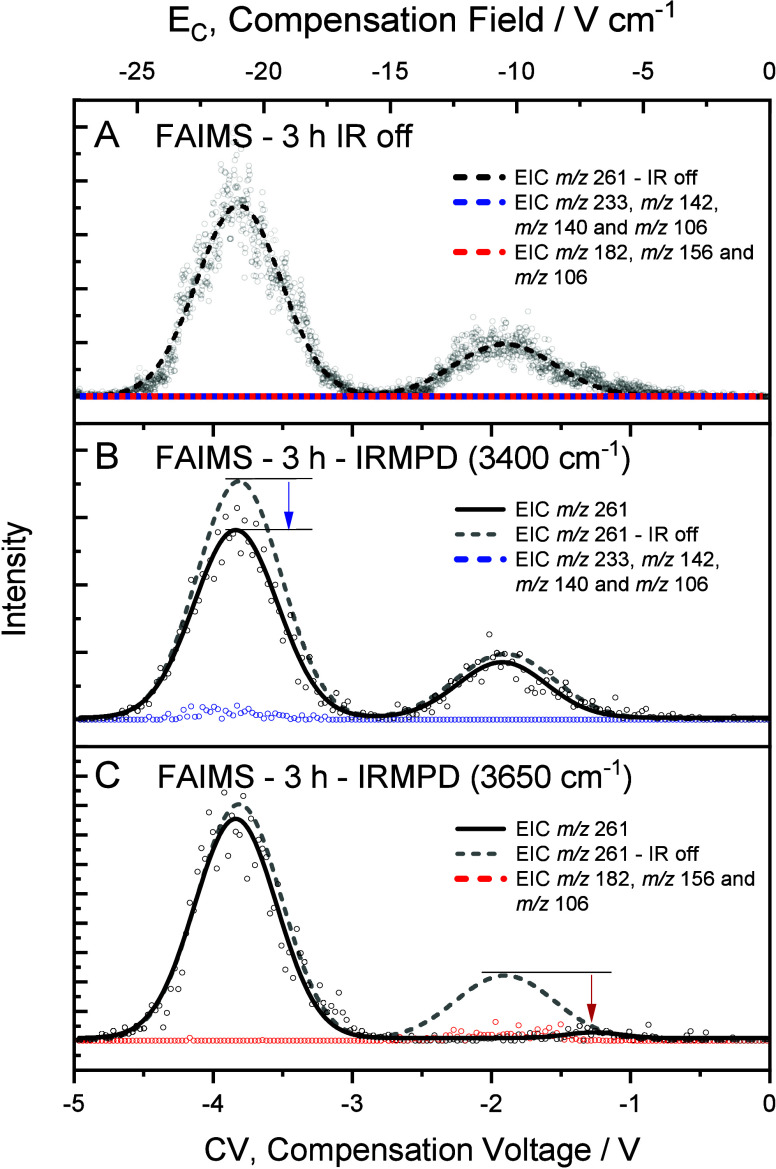
FAIMS analysis of the ion with *m*/*z* 261 for a CP solution submitted to 3 h of forced chemical. FAIMS carried out (A) without laser irradiation (B) and with 2 s of laser irradiation at 3400 cm^–1^ (absorption band of protonated CP) and (C) 3660 cm^–1^ (absorption band of the protonated TP261). The FAIMS spectra shown in (A) is normalized and represented as the dashed lines in (B) and (C) for comparison. Arrows represent the observed photodepletion at different wavenumbers.

These experiments consist of monitoring the photoinduced dissociation during the FAIMS separation at 3400 and 3660 cm^–1^, corresponding to the N---H (endocyclic) and O---H stretching bands assigned to the protonated CP and TP261 transformation product, respectively.

When the photodepletion experiment is carried out at 3400 cm^–1^ (Figure 6B), only the first population at CV −3.5 V showed a significant decrease in ion intensity and the formation of fragment ions associated with the protonated CP (Figure 6B – blue circles). When the wavenumber is set to 3660 cm^–1^ (Figure 6C), the situation inverts and the second population is almost completely depleted corresponding to the characteristic formation of TP261 fragments *m*/*z* 182, *m*/*z* 156, and *m*/*z* 106 (Figure 6C – red points).

### Control Experiments for TP261 Formation

To increase the transformation rate of cyclophosphamide so a sample with the major presence of TP261 could be obtained, a second forced chemical degradation test was carried out under the same conditions for 24 h (Figure 3C). The MS^2^ spectrum of the ions with *m*/*z* 261 after 24 h of forced degradation (Figure 3F) showed the same fragment ions when compared to the MS^2^ spectrum acquired from the sample analyzed after 3 h of heating (Figure 3E). However, as expected, the abundance of the fragment ions of the isobaric transformation product, *m*/*z* 182 and *m*/*z* 156, increased as the abundance of the ions with *m*/*z* 233, 142, and 140 decreased, demonstrating the decrease in the concentration of the prodrug as the hydrolysis reaction was carried out.

The FAIMS analysis of this same sample (Figure 4C) showed a major population of ions with *m*/*z* 261, appearing in a CV range of −2 to 0 V, with a fragmentation pattern consistent with the fragmentation pattern of the protonated transformation product TP261 observed in the previous analyses (Figure 4B), besides an almost imperceptible residual intensity of protonated CP at CV −3.5 V.

The IRMPD spectrum of the ion with *m*/*z* 261 selected from the solution maintained in forced degradation for 24 h (Figure 5C) almost exclusively showed the band corresponding to the O---H stretch at 3660 cm^–1^ in accordance with the extensive conversion of CP to TP261 expected. The IRMPD analyses of the aliquots collected after 1, 2, and 4 h of heating can be seen in Figure S2 and indicate that this variation in the intensity of the bands is progressive, suggesting a gradual increase in the concentration of the isobaric transformation product.

Therefore, the results presented here show that the presence of TP261 was effectively confirmed by the synergic use of FAIMS and IRMPD spectroscopy.

## Conclusions

This study demonstrates that the combination of high-field asymmetric waveform ion mobility spectrometry (FAIMS) and infrared multiple photon dissociation (IRMPD) spectroscopy is a powerful alternative to traditional high-performance liquid chromatography (HPLC) and reference standard dependent methods for the identification of drug degradation products.

The FAIMS, CID, and IRMPD data demonstrated that, after 3 h of forced chemical degradation by acid hydrolysis at 70 °C, there is the formation of an isobaric transformation product with *m*/*z* 261. Based on the spectral features of this species in comparison to the protonated CP spectrum, the exact nature of this transformation product could be assigned, in accordance with previous NMR reports from the literature that pointed out the presence of this species in forced degradation studies of CP. This study also demonstrates, using cyclophosphamide and its forced degradation as a model system, that the FAIMS-IRMPD approach is effective for the separation and structural elucidation of complex isobaric and, by extension, isomeric transformation products under forced degradation conditions. By eliminating the need for chromatography separations, this methodology offers a faster alternative for qualitative analysis of pharmaceutical degradation products. This work supports the broader application of FAIMS and IRMPD in stability studies and quality control, enhancing the reliability and efficiency of pharmaceutical analyses.

## Supplementary Material



## Notes

Full FAIMS spectra for the cyclophosphamide solution before and after being subjected to acid hydrolysis at 70 °C for 1, 2, 3, 4, and 24 h (Figure S1), IRMPD vibrational spectra, in the 2800 to 3800 cm^–1^ range, acquired before and after the cyclophosphamide solution was subjected to the forced chemical degradation test for 1, 2, 3, 4, and 24 h (Figure S2), other transformation products observed in the cyclophosphamide solution after 24 h of heating in the acid hydrolysis chemical degradation and their putative fragments (Table S1), and XYZ coordinates for the two geometries used to simulate the infrared absorption spectra (Table S2) (PDF)
