# Supplementary material for: Characterization of Pharmaceutical Transformation Products by High-Field Asymmetric Waveform Ion Mobility and Infrared Ion Spectroscopy Coupled to Mass Spectrometry
Source: J Am Soc Mass Spectrom. 2025 May 12;36(6):1277–85. doi: 10.1021/jasms.5c00039 (PMC12142665; doi:10.1021/jasms.5c00039)
Supplement: Supplementary file 1 [file js5c00039_si_001.pdf]

Characterization of Pharmaceutical Transformation Products by High-Field Asymmetric Waveform Ion Mobility and Infrared Ion Spectroscopy coupled to Mass Spectrometry

César A. G. Dantas, Pedro H. M. Garcia and Thiago C. Correra*
Department of Fundamental Chemistry, Institute of Chemistry, University of São Paulo
Av. Prof. Lineu Prestes, 748, Cidade Universitária, São Paulo, São Paulo, 05508-000, Brazil.
*E-mail: tcorrera@iq.usp.br

Supporting Information

Figure S1. Full FAIMS spectra acquired for the CP solution, under acid hydrolysis conditions, was heated for a) 0, b) 1, c) 2, d) 3, e) 4 and f) 24 hours. Dots indicate the extracted ion intensity for de ions with *m/z* 261……………………………..…………2

Figure S2. IRMPD vibrational spectra acquired for the CP solution, under acid hydrolysis conditions, was heated for a) 0, b) 1, c) 2, d) 3, e) 4 and f) 24 hours. Dots indicate the extracted ion intensity for de ions with *m/z* 261…………………………………………3

Table S1. Observed transformation products for the forced degradation of CP, under acid hydrolysis conditions exposed to heating for 24 hours…………………………………...4

Table S2. Optimized geometries for Cyclophosphamide and TP261……………………5

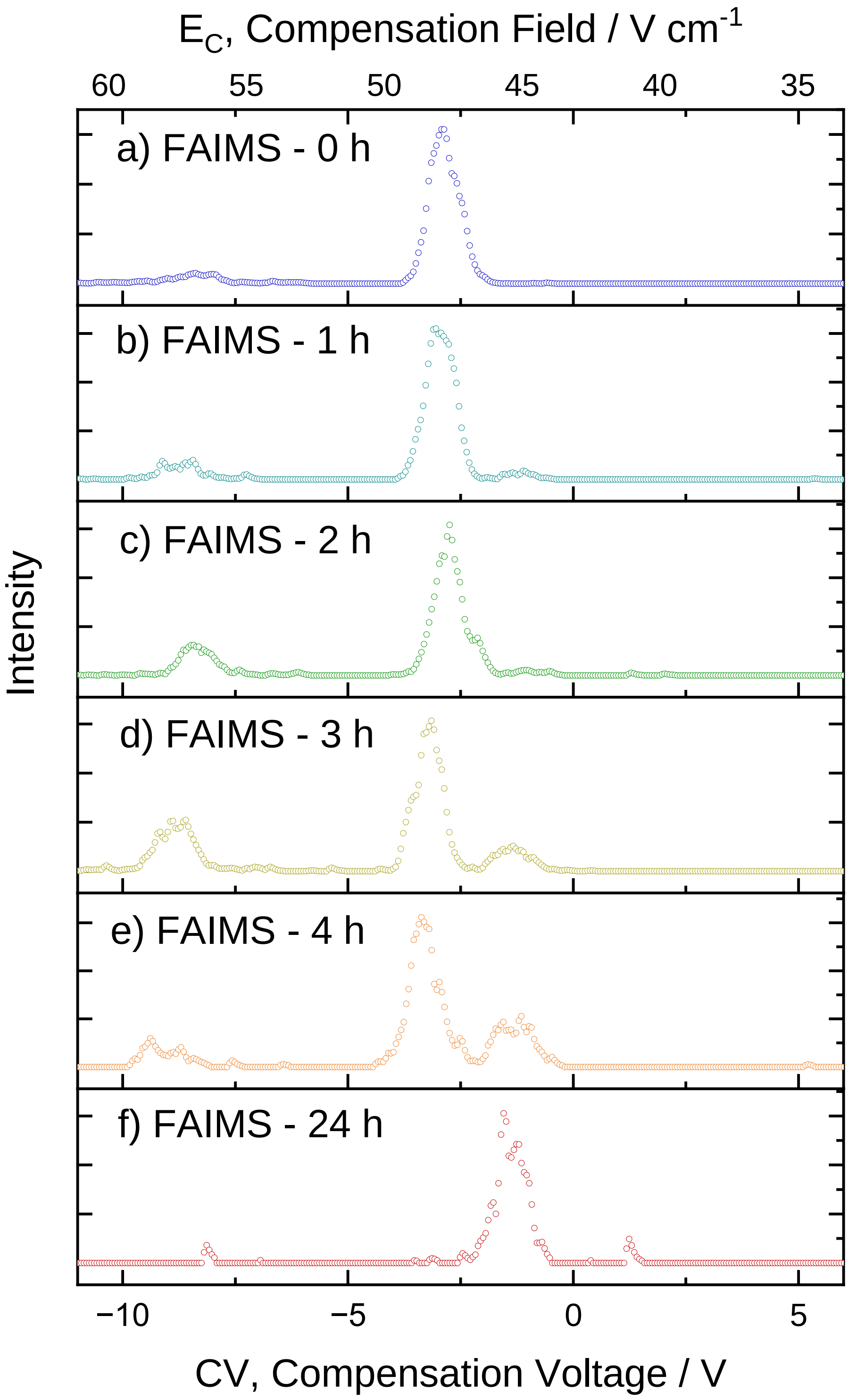

Figure S1. Full FAIMS spectra acquired for the CP solution, under acid hydrolysis conditions, was heated for a) 0, b) 1, c) 2, d) 3, e) 4 and f) 24 hours. Dots indicate the extracted ion intensity for the ions with *m/z* 261

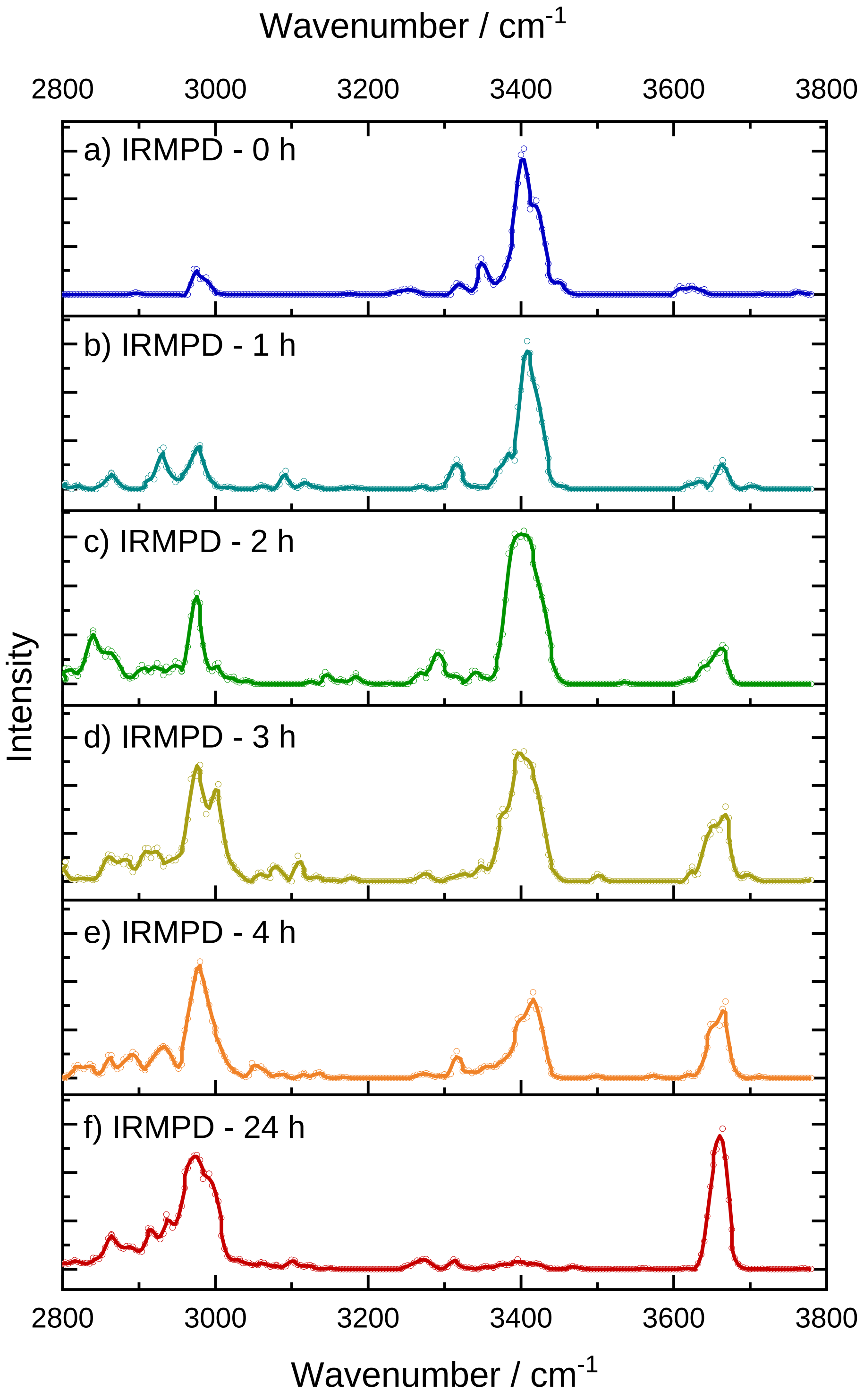

Figure S2. IRMPD vibrational spectra acquired for the CP solution, under acid hydrolysis conditions, was heated for a) 0, b) 1, c) 2, d) 3, e) 4 and f) 24 hours. Dots indicate the extracted ion intensity for the ions with *m/z* 261.

Table S1. Observed transformation products for the forced degradation of CP, under hydrolysis conditions exposed to heating for 24 hours.

**Neutral Transformation Products**	**Molecular formula and *m/z* of [M+H]$^+$**	***m/z* and Molecular formula of MS^2 fragments**
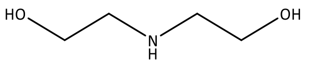	$C_4H_{12}NO_2^+$ 106	88 : $C_4H_{10}NO^+$ (-H_2O)<br>70 : $C_4H_8N^+$ (-2H_2O)
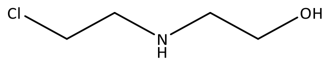	$C_4H_{11}ClNO^+$ 124	106 : $C_4H_9ClN^+$ (-H_2O)
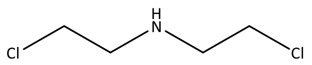	$C_4H_{10}Cl_2N^+$ 142	106 : $C_4H_9ClN^+$ (-HCl)<br>63 : $C_2H_4Cl^+$ ($C_2H_6ClN^+$)
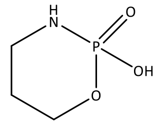	$C_3H_9NO_3P^+$ 138	110 : $CH_5NO_3P^+$ (-C_2H_4)
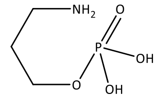	$C_3H_{11}NO_4P^+$ 156	138 : $C_3H_9NO_3P^+$ (-H_2O)<br>58 : $C_3H_8N^+$ (-H_3O_4P)
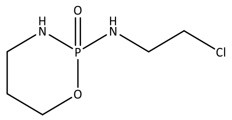	$C_5H_{13}ClN_2O_2P^+$ 199	181 : $C_5H_{11}ClN_2OP^+$ (-H_2O)<br>171 : $C_3H_9ClN_2O_2P^+$ (-C_2H_4)
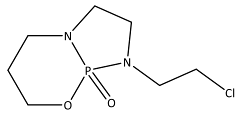	$C_7H_{15}ClN_2O_2P^+$ 225	207 : $C_7H_{13}ClN_2OP^+$ (-H_2O)
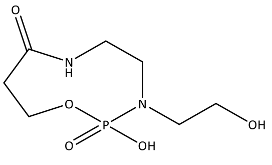	$C_7H_{16}N_2O_5P^+$ 239	221 : $C_7H_{14}N_2O_4P^+$ (-H_2O)<br>164 : $C_5H_{11}NO_3P^+$ (-C_2H_6ClN)
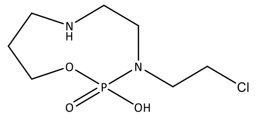	$C_7H_{17}ClN_2O_3P^+$ 243	225 : $C_7H_{15}ClN_2O_2P^+$ (-H_2O)
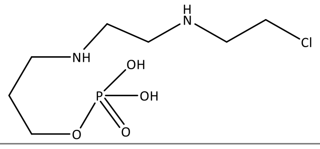	$C_7H_{19}ClN_2O_4P^+$ 261 (TP261)	182 : $C_5H_{13}NO_4P^+$ (-C_2H_6ClN)<br>156 : $C_3H_{11}NO_4P^+$ (-C_4H_8ClN)

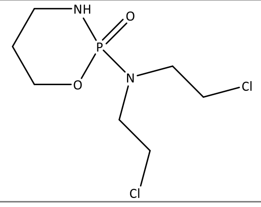	$C_7H_{16}Cl_2N_2O_2P^+$<br>261<br>(CP)	233: $C_5H_{12}Cl_2N_2O_2P^+$ ($-C_2H_4$)<br>142: $C_4H_{10}Cl_2N^+$ ($-C_3H_6NO_2P$)<br>140: $C_4H_8Cl_2N^+$ ($-C_3H_8NO_2P$)<br>106: $C_4H_9ClN^+$ ($-C_3H_7ClNO_2P$)

Table S2. Optimized geometries for Cyclophosphamide and TP261

Protonated Cyclophosphamide

17	-0.427064000	-4.145888000	-0.343030000
17	-4.132630000	1.656126000	-0.255009000
15	0.828447000	0.825466000	0.450679000
8	1.312849000	1.638613000	-0.824938000
8	0.321021000	1.939782000	1.471192000
7	-0.494627000	-0.121430000	0.314709000
7	2.109014000	-0.076013000	0.900272000
6	3.503949000	-0.003699000	0.375153000
6	3.705746000	1.208214000	-0.534521000
6	2.577512000	1.364459000	-1.538378000
6	-0.396961000	-1.429098000	-0.387983000
6	-1.875818000	0.385236000	0.569522000
6	-0.559509000	-2.604170000	0.577806000
6	-2.433913000	1.178668000	-0.613095000
1	3.704324000	-0.930567000	-0.172670000
1	4.186232000	0.044199000	1.227285000
1	3.794600000	2.128282000	0.054730000
1	4.649794000	1.085084000	-1.075462000
1	1.961182000	-0.736434000	1.655677000
1	2.707061000	2.230862000	-2.185954000
1	2.429139000	0.473311000	-2.155031000
1	0.565325000	-1.509629000	-0.898100000
1	-1.170248000	-1.466635000	-1.160107000
1	-1.875420000	0.994667000	1.472377000
1	-2.501993000	-0.488070000	0.762779000
1	0.221989000	-2.609717000	1.340018000
1	-1.532933000	-2.600356000	1.070263000
1	-1.867555000	2.096136000	-0.784654000
1	-2.447126000	0.589282000	-1.531827000
1	0.765999000	2.800824000	1.421212000

Protonated TP261

17	-57.527220000	10.672446000	-86.323394000
6	-58.035886000	8.928983000	-86.300289000
6	-59.453091000	8.773482000	-86.824955000
7	-59.564973000	9.312043000	-88.225100000
6	-60.973938000	9.373241000	-88.736813000
6	-61.071592000	9.756035000	-90.223491000
7	-60.731051000	8.637160000	-91.180316000

6	-61.820892000	7.588699000	-91.308680000
6	-61.427322000	6.399586000	-92.201374000
6	-60.702567000	5.243564000	-91.499479000
8	-59.357778000	5.571651000	-91.114572000
15	-58.992629000	6.243844000	-89.686361000
8	-57.684056000	5.448898000	-89.197829000
8	-58.577884000	7.716158000	-90.041264000
8	-60.105623000	6.152878000	-88.682687000
1	-59.006453000	8.664486000	-88.889567000
1	-56.958361000	5.430147000	-89.843475000
1	-61.264074000	4.918590000	-90.615763000
1	-60.616228000	4.401625000	-92.188172000
1	-62.359383000	5.987669000	-92.608073000
1	-60.835226000	6.741337000	-93.060390000
1	-62.045453000	7.246989000	-90.295537000
1	-62.696839000	8.107699000	-91.708039000
1	-59.776805000	8.200856000	-90.837657000
1	-60.398278000	10.590366000	-90.447937000
1	-62.089529000	10.096334000	-90.433300000
1	-61.412221000	8.392671000	-88.537269000
1	-61.509804000	10.127696000	-88.152427000
1	-59.133196000	10.243018000	-88.240547000
1	-59.734362000	7.714963000	-86.872992000
1	-60.163120000	9.316982000	-86.193287000
1	-57.304153000	8.373089000	-86.892897000
1	-57.992047000	8.592063000	-85.264521000
1	-60.559277000	9.041482000	-92.105937000
